# Fusion of the *HMGA2* and *C9orf92* genes in myolipoma with t(9;12)(p22;q14)

**DOI:** 10.1186/s13000-016-0472-8

**Published:** 2016-02-09

**Authors:** Ioannis Panagopoulos, Ludmila Gorunova, Antonio Agostini, Ingvild Lobmaier, Bodil Bjerkehagen, Sverre Heim

**Affiliations:** Section for Cancer Cytogenetics, Institute for Cancer Genetics and Informatics, The Norwegian Radium Hospital, Oslo University Hospital, P.O. Box 4953, Nydalen, NO-0424 Oslo, Norway; Centre for Cancer Biomedicine, Faculty of Medicine, University of Oslo, Oslo, Norway; Department of Pathology, The Norwegian Radium Hospital, Oslo University Hospital, Oslo, Norway; Faculty of Medicine, University of Oslo, Oslo, Norway

**Keywords:** Myolipoma, Chromosome translocation, Fusion gene, *HMGA2*, *C9orf92*

## Abstract

**Background:**

Myolipoma of soft tissue is an extremely rare benign tumor composed of mature adipose tissue and smooth muscle cells. It is found predominantly in women. The cytogenetic and molecular genetic features of myolipomas remain largely unexplored. Here we present the first cytogenetically analyzed myolipoma.

**Methods:**

Cytogenetic and molecular genetic analyses were done on a myolipoma.

**Results:**

G-banding analysis of short-term cultured cells from the myolipoma yielded a karyotype with a single clonal chromosome abnormality: 46,XX,t(9;12)(p22;q14). Fluorescence in situ hybridization experiments demonstrated that *HMGA2* (in 12q14) was rearranged. Molecular genetic analysis showed that the translocation resulted in fusion of *HMGA2* with the *C9orf92* gene (from 9p22). The *HMGA2-C9orf92* fusion transcript would code for a putative protein containing amino acid residues 1–94 of HMGA2 and 6 amino acid residues from the out-of-frame fusion with exon 4 of *C9orf92*.

**Conclusion:**

The pattern of *HMGA2* rearrangement in the present case of myolipoma is similar to what is found in other benign connective tissue tumor types, including lipomas, i.e., disruption of the *HMGA2* locus leaves intact exons which encode the AT-hook domains but separates them from the 3´-terminal part of the gene. Whether any genetic features differentiate myolipomas from regular lipomas with *HMGA2*-involvement is a question that cannot be answered until more cases of the former tumor type are subjected to genetic analysis.

**Electronic supplementary material:**

The online version of this article (doi:10.1186/s13000-016-0472-8) contains supplementary material, which is available to authorized users.

## Background

Myolipoma of soft tissue is a benign tumor composed of mature adipose tissue and smooth muscle [1]. It was first described as an entity in 1991 by Meis and Enzinger (as myolipoma of soft tissue) [2] and Scurry et al. (as soft tissue lipoleiomyoma) [3]. The tumor is extremely rare and is found predominantly in women. Searching PubMed (http://www.ncbi.nlm.nih.gov/pubmed/) using the term “myolipoma” we found that around 50 cases have been reported since 1991 (Additional file 1: Table S1; data updated October 20, 2015). These tumors were found in 35 females and 14 males. The median age was 48 years (range, 4–83) (Additional file 1: Table S1). Most publications have described single cases, further testifying to the rarity of the disease. The tumor is usually found as a deep-seated mass within the abdominal cavity, retroperitoneum, or inguinal region [2, 4–8], although other locations have been reported such as the tongue base, mesentery, pericardium, and eyelid (Additional file 1: Table S1). No cases with focal recurrence, metastatic disease or other signs of malignant transformation have been reported and cure is achieved by surgical resection [1].

The cytogenetic and molecular genetic features of myolipomas remain largely unexplored. In the 2013 edition of “WHO classification of tumours of soft tissue and bone”, the only genetic information on these tumors is that expression of full-length *HMGA2* was detected by RT-PCR in myolipoma of the pelvic cavity [1, 9].

Here we present the first cytogenetically analyzed myolipoma. The tumor had a t(9;12)(p22;q14) as the sole karyotypic aberration resulting in fusion of *HMGA2* with the *C9orf92* gene.

## Methods

### Ethical approval

The study was approved by the Regional Committee for Medical and Health Research Ethics, South-East Norway (REK Sør) http://helseforskning.etikkom.no). Written informed consent was obtained from the patient. The consent included acceptance that his clinical details be published. The ethics committee’s approval included a review of the consent procedure. All patient information has been anonymized.

### Patient

**Fig. 1:**
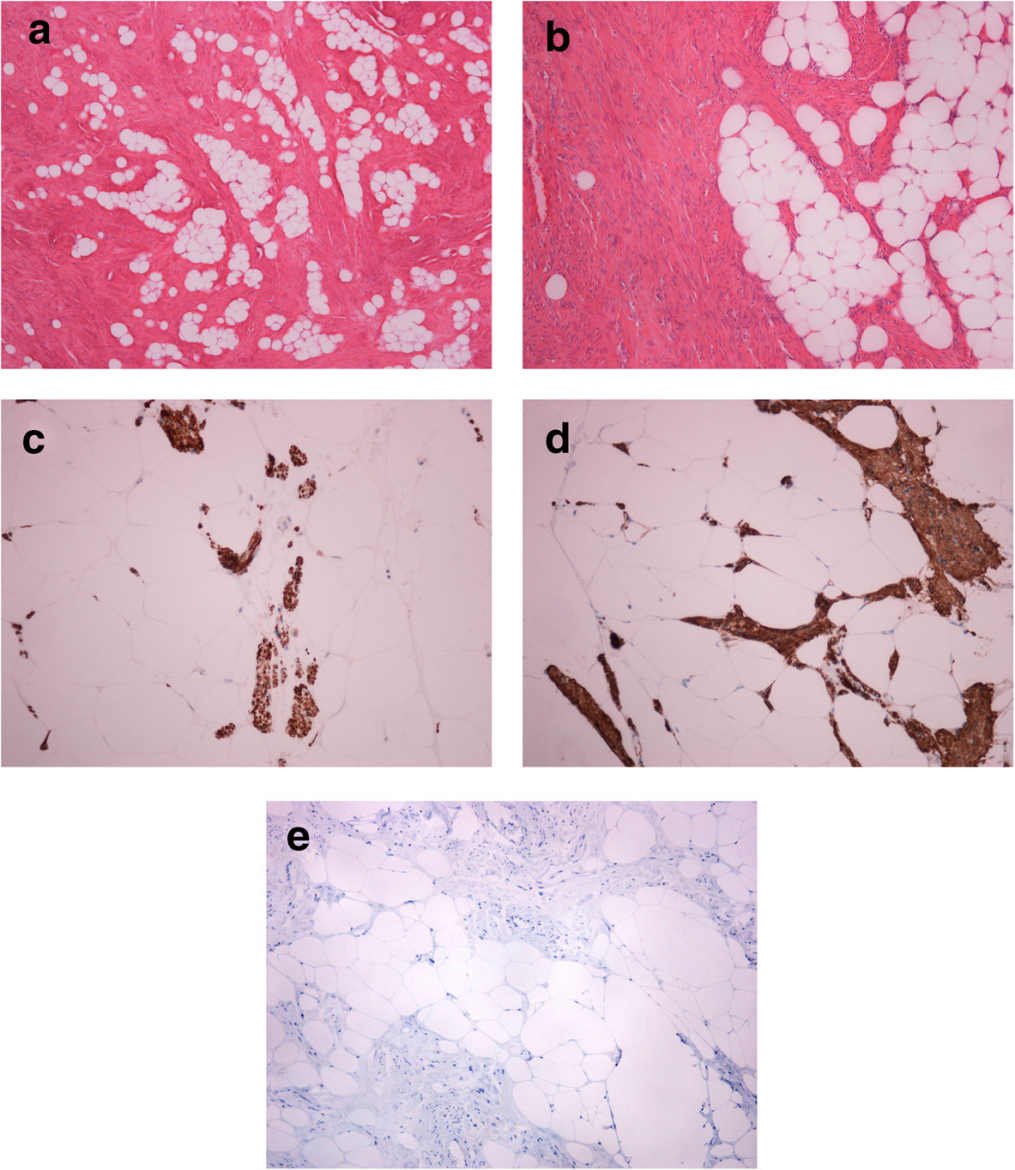
Microscopical image of the myolipoma. **a** and **b**) H&E stained slides 10X (**a**) and 20X (**b**) magnification with mature fat between bundles of spindle cells, confirmed as smooth muscle fibers by immunohistochemical examination showing positive reaction for desmin (**c**) and smooth muscle actin (SMA) (**d**). MDM2 immunostaining was negative (**e**)

The patient is a 66 years old female who underwent an abdominal CT-scan due to pain radiating into the left lower extremity. The scan revealed a well demarcated, lipogenic tumor in the left retroperitoneum measuring almost 20 cm in greatest diameter, invoking a suspicion of liposarcoma. The tumor was completely excised. The operation specimen showed a lipomatous tumor which was macroscopically well demarcated and without infiltrative growth. Microscopic evaluation showed a lipomatous tumor with areas with smooth muscle differentiation, without signs of malignancy (Fig. 1a and b). There were no suspect lipoblasts. Smooth muscle fibers showed positive reaction for desmin (Fig. 1c) and smooth muscle actin (SMA) (Fig. 1d) by immunohistochemical examination. MDM2 immunostaining was negative (Fig. 1e).

### Chromosome banding analysis and Fluorescence in situ hybridization (FISH)

Fresh tissue from a representative area of the tumor was received and cells from it were short-term cultured and analyzed cytogenetically as part of our diagnostic routine as described elsewhere [10]. The karyotype was written according to the International System for Human Cytogenetic Nomenclature (ISCN) 2013 guidelines [11].

**Fig. 2:**
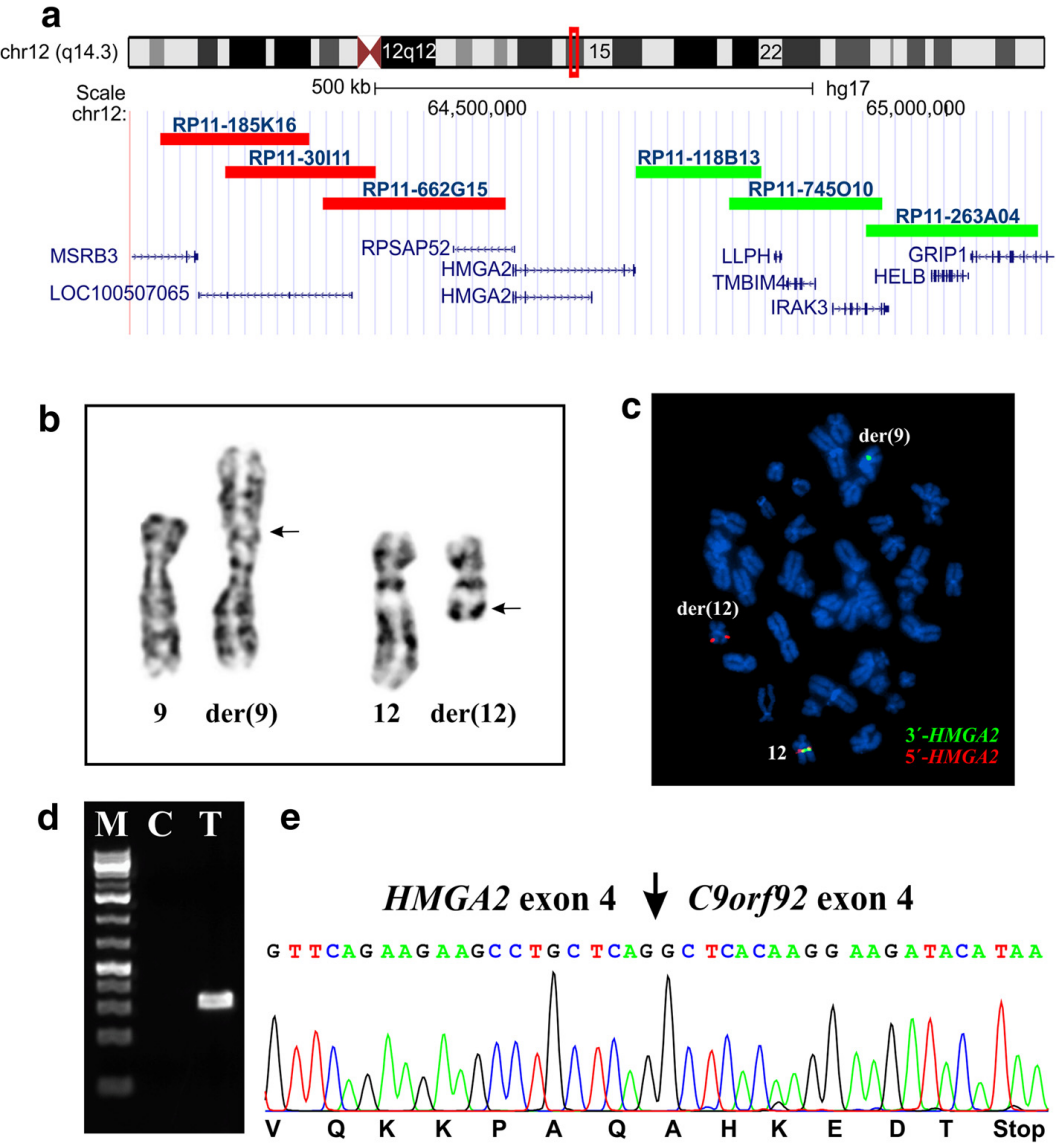
Cytogenetic, FISH, and RT-PCR analyses of the myolipoma. **a**) Chromosome 12 ideogram showing the location of the *HMGA2* locus and the BACs used for FISH experiments. The investigated region is indicated as a red box. **b**) Partial karyotype showing the der(9)t(9;12)(p22;q14) and der(12)t(9;12)(p22;q14) together with the corresponding normal chromosome homologs; breakpoint positions are indicated by arrows. **c**) FISH analysis with an *HMGA2* breakapart probe. The green signal (pool of the BACs RP118B13, RP11-745O10, and RP11-263A04) is moved to der(9) whereas the red signal (pool of the BACs RP11-185K16, RP11-30I11, and RP11-662G15) is seen on der(12). **d**) Amplification of an *HMGA2-C9orf92* cDNA fragment using primers HMGA2-936F1 and C9orf92-316R1 in myolipoma (T) but not in control (C). M, 1 Kb DNA ladder (GeneRuler, Fermentas), Bl, Blank, water in cDNA synthesis. **e**) Partial sequence chromatogram of the cDNA fragment showing the fusion of *HMGA2* with *C9orf92*

FISH analysis based on the karyotyping findings (see below) was performed on metaphase plates as described previously [10]. BAC clones were retrieved from the Human genome high-resolution BAC re-arrayed clone set (the “32k set”; BACPAC Resources, http://bacpac.chori.org/pHumanMinSet.htm). The “32k set” is mapped on the UCSC Genome browser on Human May 2004 (NCBI/hg17) assembly. Mapping data for the 32k human re-array are available in an interactive web format (http://bacpac.chori.org/pHumanMinSet.htm, from the genomic rearrays page ) and are obtained by activation of the ucsc browser track for the hg17 UCSC assembly from the “32k set” homepage (http://bacpac.chori.org/genomicRearrays.php). The BAC clones were selected according to physical and genetic mapping data on chromosome 12 as reported on the Human Genome Browser at the University of California, Santa Cruz website (May 2004, http://genome.ucsc.edu/). In addition, FISH mapping of the clones on normal controls was performed to confirm their chromosomal location. The clones used were RP11-185 K16, (chr12:64103524–64274514), RP11-30I11 (chr12:64178505–64349708), RP11-662G15 (chr12:64288763–64498219), RP118B13 (chr12:64644968–64789255), RP11-745O10 (chr12:64752327–64926193), and RP11-263A04 (chr12:64908453–65103538). All of them map to chromosome subband 12q14.3 (Fig. 2a). DNA was extracted, and probes were labelled with Fluorescein-12-dCTP (PerkinElmer, Boston, MA, USA) and Texas Red-5-dCTP (PerkinElmer) in order to obtain green and red signals, respectively, using the Abott’s nick translation kit (Des Plaines, IL, USA), and hybridized according to Abbott Molecular recommendations (http://www.abbottmolecular.com/home.html). A homemade breakapart *HMGA2* probe was used. The 5´-end of the probe (red signal) was constructed from a pool of the clones RP11-185K16, RP11-30I11, and RP11-662G15. The 3´-end of the probe (green signal) was constructed from a pool of the clones RP118B13, RP11-745O10, and RP11-263A04. All of them map to chromosome subband 12q14.3 and cover the *HMGA2* locus (Fig. 2a).

### Molecular analyses

Tumor tissue adjacent to that used for cytogenetic analysis and histologic examination had been frozen and stored at −80 °C. Total RNA was extracted using miRNeasy Mini Kit according to the manufacturer’s instructions (Qiagen Nordic, Oslo, Norway). Tumor tissue was disrupted and homogenized in Qiazol Lysis Reagent (Qiagen) using 5 mm stainless steel beads and TissueLyser II (Qiagen). Subsequently, total RNA was purified using QIAcube (Qiagen). Human Universal Reference Total RNA was used as control (Clontech Laboratories, TaKaRa-Bio Group, Europe/SAS, Saint-Germain-en-Laye, France). According to the company’s information, it is a mixture of total RNAs from a collection of adult human tissues chosen to represent a broad range of expressed genes. Both male and female donors are represented.

The 3´-RACE methodology was described in detail elsewhere [10]. To verify the results obtained by 3´-RACE, i.e., the presence of an *HMGA2-C9orf92* chimeric transcript (see below), RT-PCR was performed using the forward HMGA2-936F1 (5´-AGC CCT CTC CTA AGA GAC CCA G-3´) and the reverse C9orf92-316R1 (5´-TGA AGT TTT AAT CAA CAC AAG CAG C-3´) primers. Total RNA (1 μg) was reverse-transcribed in a 20 μL reaction volume using iScript Advanced cDNA Synthesis Kit for RT-qPCR according to the manufacturer’s instructions (Bio-Rad Laboratories, Oslo, Norway). The 25 μL PCR volume contained 12.5 μL Premix Ex Taq DNA Polymerase Hot Start Version (Takara Bio), 1 μL of cDNA, and 0.4 μM of each of the forward HMGA2-936F1 and reverse C9orf92-316R1 primers. PCR amplifications were run on a C-1000 Thermal cycler (Bio-Rad Laboratories) with an initial denaturation at 94 °C for 30 s, followed by 35 cycles of 7 s at 98 °C, 30 s at 58 °C, 1 min at 72 °C, and a final extension for 5 min at 72 °C. Three μL of the PCR products were stained with GelRed (Biotium, Hayward, CA, USA), analyzed by electrophoresis through 1.0 % agarose gel, and photographed. The remaining 22 μL PCR products were purified using the MinElute PCR purification kit (Qiagen) and sequenced at GATC Biotech (Germany, http://www.gatc-biotech.com/en/home.html). The BLAST software (http://blast.ncbi.nlm.nih.gov/Blast.cgi) was used for computer analysis of sequence data.

## Results

### G-banding analysis

G-banding analysis of short-term cultured cells from the myolipoma yielded a karyotype with a single clonal chromosome abnormality: 46,XX,t(9;12)(p22;q14) [12] (Fig. 2b).

FISH experiments showed that the *HMGA2* probe was split with one signal on der(12) and the other on der(9) (Fig. 2c).

### Molecular genetic analysis

3´-RACE analysis amplified a single fragment (Fig. 2d). Subsequent Sanger sequencing showed that it was a chimeric cDNA fragment in which exon 4 of *HMGA2* from 12q14 (nt 1093 in the reference sequence with accession number NM_003483.4) was fused to exon 4 of the *C9orf92* gene from 9p22 (nt 261 in the reference sequence NM_001271829.1).

PCR with the primers HMGA2-936F1 and C9orf92-316R1 amplified a cDNA fragment from myolipoma but not from control (Fig. 2d). Direct sequencing of the PCR product showed the same fusion breakpoint as that detected in the 3´-RACE amplified fragment (Fig. 2e).

## Discussion

We describe the first cytogenetic and molecular genetic analysis of a myolipoma. The tumor had an acquired chromosomal translocation, t(9;12)(p22;q14), which resulted in fusion of the *C9orf92* (from 9p22) and *HMGA2* (from 12q14) genes. The *HMGA2-C9orf92* fusion transcript codes for a putative protein which contains amino acid residues 1–94 of HMGA2 protein (accession number NP_003474.1), corresponding to exons 1–4 of the gene, and 6 amino acid residues (AHKEDT) coming from an out-of-frame fusion with exon 4 of *C9orf92*.

Information on C9orf92 is very scant and nothing is known about its cellular localization or function (http://www.ncbi.nlm.nih.gov/gene/100129385). Two transcript variants have been reported: Transcript variant 1 (reference sequence NM_001271829) which represents the shorter transcript and encodes a functional protein, and transcript variant 2 (reference sequence NR_073471) which uses an alternative 5' exon structure compared to variant 1. Transcript variant 2 is a non-coding RNA due to the presence of an upstream open reading frame (ORF) that is predicted to interfere with translation of the longest ORF (http://www.ncbi.nlm.nih.gov/gene/100129385). *C9orf92* is low expressed in many normal human tissues as shown by RNA-sequencing (http://www.genecards.org/cgi-bin/carddisp.pl?gene=C9orf92&keywords=c9orf92).

The translocation t(9;12)(p22;q14 ~ 15), or variants thereof, was reported before in lipomas [12–15], uterine leiomyomas [16, 17], chondroid hamartomas [18], and pleomorphic adenomas [19]. In lipomas and pleomorphic adenomas, a cytogenetically similar recombination between 9p and 12q was shown to result in the fusion of *HMGA2* with *NFIB* [12, 13, 15, 19] which maps 2.2 Mbp distal to *C9orf92.*

In lipomas, two different *HMGA2–NFIB* fusion transcripts have been identified. In the first type, exons 1–4 of *HMGA2* were fused to exon 9 of *NFIB* [12, 13, 15]. In the second type, the fusion transcript consisted of the first three exons of *HMGA2*, exon 6 of *MSRB3* (12q14.3), and exon 9 of *NFIB* [15]. In both transcript types, a stop codon is present on the 3′ side shortly after the fusion point of *HMGA2* with *NFIB* or *MSRB3* [12, 13, 15]. Similarly, the *HMGA2-NFIB* fusions found in pleomorphic adenomas also contain a stop codon located near but downstream of the fusion point [19].

The *NFIB* gene codes for a transcription factor which recognizes and binds the palindromic sequence 5-TTGGCNNNNNGCCAA-3 of viral and cellular promoters (http://www.genecards.org/cgi-bin/carddisp.pl?gene=NFIB&keywords=NFIB). No functional information is at hand about the *C9orf92* gene (http://www.genecards.org/cgi-bin/carddisp.pl?gene=C9orf92&keywords=c9orf92).

Although the t(9;12)(p22;q14 ~ 15) thus appears to be heterogeneous at the molecular level, generating *HMGA2-NFIB* in pleomorphic adenoma and lipomas but *HMGA2-C9orf92* in the only myolipoma examined, the pathogenetic pattern behind these changes is similar to that of *HMGA2* rearrangements found generally in benign connective tissue tumors, i.e., disruption of the *HMGA2* locus leaving intact exons 1–3 which encode the AT-hook domains and separates them from the 3´-untranslated region of the gene (3´-UTR) [20]. The 3´-UTR of *HMGA2* was shown to regulate the transcription of the *HMGA2* gene [21, 22].

## Conclusion

The present case of myolipoma underscores the frequent and general role of *HMGA2* rearrangements in the genesis of several benign connective tissue tumors. Further studies are necessary to find out whether myolipomas of soft tissue in any systematic way differ from the other tumor types in the exact manner in which *HMGA2* is abrogated, in particular whether fusion with *C9orf92* is a general feature of these rare neoplasms.

## Additional file

Additional file 1: Table S1. Cases of myolipomas which have been reported from 1991 until October 20, 2015. They were found searching pubmed (http://www.ncbi.nlm.nih.gov/pubmed/) using the term “myolipoma”. (XLSX 17 kb)

## Abbreviations

CT: Computed tomography
FISH: Fluorescence in situ hybridization
ORF: Open reading frame
PCR: Polymerase chain reaction
RACE: Rapid amplification of cDNA ends
UTR: Untranslated region
WHO: World Health Organization
